# A randomized trial to evaluate the effectiveness of an individual, education-based safe transport program for drivers aged 75 years and older

**DOI:** 10.1186/1471-2458-13-106

**Published:** 2013-02-05

**Authors:** Lisa Keay, Kristy Coxon, Julie Brown, Elizabeth Clarke, Soufiane Boufous, Anita Bundy, Serigne Lo, Rebecca Ivers

**Affiliations:** 1 The George Institute for Global Health, PO Box M201, Missenden Rd, Camperdown, NSW, 2050, Australia; 2 Sydney Medical School, The University of Sydney, Paramatta Rd, Sydney, NSW, 2006, Australia; 3 Sydney School of Public Health, The University of Sydney, Paramatta Rd, Sydney, NSW, 2006, Australia; 4 Neuroscience Research Australia, Barker St, Randwick, NSW, 2031, Australia; 5 University of New South Wales, Sydney, Australia; 6 Kolling Institute, Building 6 Level 10, RNS Hospital, St Leonards, NSW, 2065, Australia; 7 Transport and Road Safety (TARS) Research, Level 1, Main old building, The University of New South Wales Sydney, Sydney, NSW, 2052, Australia; 8 Faculty of Health Sciences, University of Sydney, PO Box 170, Lidcombe, NSW, 2041, Australia

**Keywords:** Driving, Ageing, Naturalistic driving, Participation, Mobility, Safety, Road traffic injury, Education

## Abstract

**Background:**

There are concerns over safety of older drivers due to increased crash involvement and vulnerability to injury. However, loss of driving privileges can dramatically reduce independence and quality of life for older members of the community. The aim of this trial is to examine the effectiveness of a safe transport program for drivers aged 75 years and older at reducing driving exposure but maintaining mobility.

**Methods and design:**

A randomised trial will be conducted, involving 380 drivers aged 75 years and older, resident in urban and semi-rural areas of North-West Sydney. The intervention is an education program based on the Knowledge Enhances Your Safety (KEYS) program, adapted for the Australian context. Driving experience will be measured objectively using an in-vehicle monitoring device which includes a global positioning system (GPS) to assess driving exposure and an accelerometer to detect rapid deceleration events. Participation will be assessed using the Keele Assessment of Participation (KAP). Data will be analysed on an intention-to-treat basis; the primary outcomes include driving exposure, rapid deceleration events and scores for KAP. Secondary outcomes include self-reported measures of driving, socialisation, uptake of alternative forms of transport, depressive symptoms and mood. A detailed process evaluation will be conducted, including examination of the delivery of the program and uptake of alternative forms of transport. A subgroup analysis is planned for drivers with reduced function as characterized by established cut-off scores on the Drivesafe assessment tool.

**Discussion:**

This randomised trial is powered to provide an objective assessment of the efficacy of an individually tailored education and alternative transportation program to promote safety of older drivers but maintain mobility. Trial registration: Australian New Zealand Clinical Trials Registry ACTRN12612000543886.

## Background

Older people are a large and growing sector of the driving population. Concerns over safety of older drivers have been raised due to increased crash involvement and vulnerability to crash injury [1-3]. Crash involvement per mile driven and likelihood for driver responsibility begins to increase from age 65 [4] and by age 85 likelihood of crash involvement is approximately 2.5 times higher than that of the younger drivers [5]. However, concerns over safety need to be tempered by the fact that driving is an important means to maintain independence and community participation for older people. Loss of driving privileges has been linked to depression and early admission to residential care [6].

Unlike younger drivers whose higher crash rate is attributed to inexperience and risky driving behaviour [4] the high crash rate for older drivers is explained by a different set of factors including visual, cognitive and functional decline, chronic conditions and medication. Compared with younger drivers, older drivers are over-involved in angle crashes, overtaking or merging crashes, and especially intersection crashes [4,7-9].

Driver licensing systems regulate driving privileges and most jurisdictions have restricted licenses for drivers with functional limitations. These are designed to keep less competent drivers in low-risk driving situations. Screening programs to evaluate functional status have been pilot tested in Australia [10] and proposed in the US [11]. However, recent reviews of available evidence find that there was no off-road screening test of fitness to drive that could be justified as a determinant of licensing status.[12,13] This was mainly due to concern about cost-effectiveness of screening all older drivers and limited effectiveness at identifying high risk drivers.

Self- regulation remains a central strategy for reducing crash risk amongst older drivers. Approximately 3-5%[14,15] of older drivers retire from driving each year and one quarter to one third make at least one adaptation to the way they drive.[16-18] Self-restriction and giving up driving has consistently been linked to decline in vision,[15,17,19-23] cognition [17,19,22,24,25], physical strength[24] and poor health [15,17,21,22,25,26]. Importantly, those drivers who self-regulate their driving have been shown to have poorer performance in on-road assessment.[18] An analysis of a case series of fatal crashes involving older drivers from the North American Fatality Analysis Rating System (FARS-2003) found that those who drive in the daylight were 28% (8 am-1 pm) and 37% (2 pm-8 pm) less likely to be injured in a crash [27]. Older drivers with a previous motor vehicle conviction, are 35% less likely to be injured in a crash presumably as these individuals acknowledge their limitations and take corrective action such as reducing their exposure to driving[27]. This epidemiologic evidence provides support for the use of self-restriction as a means to optimise the safety of older drivers.

While self-regulation is a promising tool to promote safety, external factors may over-ride considerations of driving ability and prevent an informed and timely decision [27]. It has been shown that a personal preference to be the driver (rather than a passenger) keeps drivers on the road [28]. The availability of alternate transportation[16] and another driver in the household [22] also increases the likelihood of stopping driving or limiting driving. Surveys find few current drivers have planned for retirement from driving [16].

We hypothesise that self-regulation can be optimised and propose to evaluate the effectiveness of an integrated program consisting of education about safe driving and alternative transportation, on driving exposure and safety. In recognition that loss of driving can negatively impact independent mobility, we will evaluate the impact on community participation and socialisation. Lastly, the cost-effectiveness of the program will be evaluated and provide important direction for policy makers and guide decisions about programs for older drivers to manage their driving and resource allocation to transportation schemes for older members of the community.

## Methods

**Figure 1:**
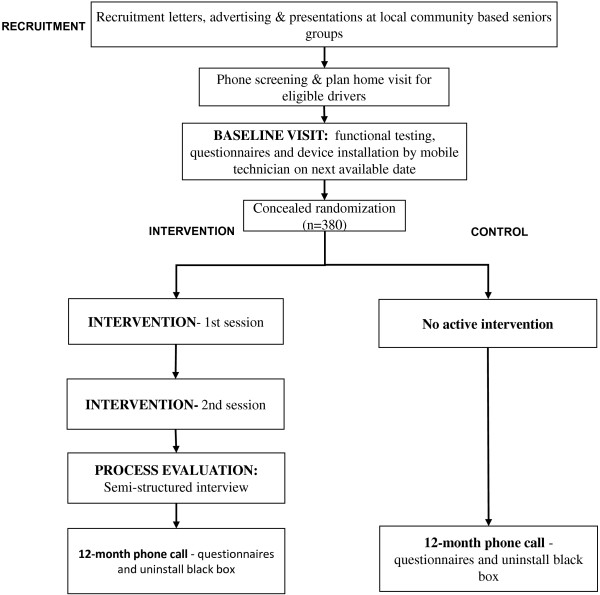
Flow chart of participation in the randomized trial evaluating a safe transport program.

The study is a randomised trial, designed in line with the CONSORT statement [29], involving 380 drivers. As the greatest population of older drivers in Australia is in outer suburbs and semi-rural areas [30], we will recruit licensed drivers’ aged 75 years and older, resident in two local government areas (LGA) on the suburban outskirts of Sydney, the Hills and Hornsby-Kuringai Shires. These areas were selected as they have greater than the national population median of seniors resident in the LGA (>17% aged 65 and over) and low availability of public transportation as defined by absent rail and irregular or no public bus services. Participants will be recruited through advertising in local media, seniors groups and through a mail-out to members of a motoring organisation in New South Wales (Figure 1).

In late 2009 the investigators conducted a series of 3 focus groups with older drivers, who were members of a community-based seniors club in the Hills Shire. There was consensus that driving was essential to their independence, with participants citing lack of transport alternatives with no taxi service, sporadic private bus transport and strong personal preference for being able to continue driving. As reported elsewhere [16] few had planned for retirement from driving though loss of driving privileges was perceived as incompatible with continued living in this area. All participants felt there were significant safety issues for older drivers but that these issues were limited to only a few drivers, not well represented in the media and overestimated by the driving public. This consultation highlighted the importance of independent mobility and the need for objective data to inform policy in this area.

The study will be conducted in adherence with the Declaration of Helsinki and all participants will sign a record of informed consent. The study protocol has been approved by the University of Sydney Human Research Ethics Committee.

### Study procedures

Drivers interested in participating will be asked to contact the study centre by phone and complete a short interview to confirm eligibility. Participants are limited to residents of the Hills and Hornsby-Kuringai Shires so local transport services can be integrated into our intervention program. The program is only available in English and requires involvement of the participant in the education and planning so participants who do not have conversational English and those with evidence of a significant cognitive deficit will be excluded from the study [31]. As driving will be measured objectively, we require that participants are the primary driver of their own car and that they agree to have the in-vehicle monitoring device installed for a period of 12 months. The eligibility criteria are therefore; aged 75 years or older and resident in the Hills Shire, holder of a current drivers’ license, primary driver of their own vehicle, speak conversational English and two or less errors on the Short Portable Mental Status Questionnaire [31].

A baseline assessment will characterise the drivers enrolled in this study and will be conducted during a home visit lasting 1.5-2 h. Comorbidities [32], medication and alcohol use, presence of depressive symptoms (Geriatric Depression Scale [33]) and mood (Positive and Negative Affect Scale [34]) will be assessed by questionnaire. Driving relies heavily on cues from vision and we will assess vision using a measure of contrast sensitivity (Mars Perspectix letter chart) [35]. Contrast sensitivity appears to be a better predictor of driving performance than high contrast visual acuity. Owsley et al. [36] showed that contrast sensitivity was associated with crash involvement whereas visual acuity and glare sensitivity showed no significant associations. There are a number of measures of cognition relevant to driving abilities [37] and the Trail Making Test Part B is selected as it is portable, easy to administer, normative data are available [38] and it is predictive of driving performance [39]. The Trail Making Test Part B is a test of visual scanning and processing and executive function [40]. Longer times to complete the TMT Part B have been associated with poor driving performance in a number of studies [41-44].

Use of spectacles, problems with vision including cataracts and how recently they had an eye examination will be recorded. Visual attention implies the ability to pay attention to more than one object in the visual field and deficits in visual attention are related to driving errors [45,46] and crash risk [47] in older drivers. There are a number of assessments which evaluate visual attention however, we elected to administer the DriveSafe assessment tool as it assesses visual attention in a series of driving scenes and also considers awareness of functional limitations [48]. DriveSafe produces an overall score and has also been validated against on-road driving performance [49].

### Randomisation

Each individual will be allocated to receive the intervention or form part of the control group after completion of the baseline assessment. Treatment will be assigned via remote login to the on-line study database which has an inbuilt randomisation feature [50]. The randomisation sequence available through the on-line system was generated with random block sizes using an on-line randomisation plan generator by an investigator not involved in baseline assessments (LK) and will remain concealed.

### Intervention

We propose to work with a group of older drivers to help them make informed choices about transport, thereby promoting safety but maintaining mobility and community engagement. Educational programs have been shown to increase use of safe driving strategies [51] and have included classroom modules [52], one-on-one counselling [53], video programs [54] and home based CD-rom or workbooks [6]. The KEYS program was selected as the basis for our intervention as it is well grounded in social-cognitive theories of behaviour [53]. While not proven to be protective against crash risk in a small trial in the US [55], Owsley demonstrated that the KEYS educational program, administered to a group of older drivers with visual acuity deficits or slowed visual speed of processing, increased self-reported safe driving practices [56].

The KEYS program has been adapted for the Australian setting and comprises two one-on-one sessions conducted in the participant’s home. The education is not ‘one size fits all’, instead it will be customised to meet the stage of behaviour change of the participant using the Precaution Adoption Process Model [57]. Understanding the stage of precaution adoption and self-regulation behaviour of each individual will allow the educator to tailor the approach and educational content. This targeted education aims to move participants through the stages of behaviour change towards adoption of safer driving practices [58].

The skill-building component raises the drivers’ awareness of their own skills and abilities and helps drivers match their driving skills to their driving exposure. The educator assists drivers translate this information into appropriate self-regulatory strategies that aim to promote safe driving for as long as possible. Finally, the intervention will build participant’s confidence in their ability to make changes to their driving habits. Strategies will include giving examples of other older drivers who have changed the way they drive and working with participants to set goals regarding changing their driving.

We know that lack of alternative transportation keeps older drivers on the road, independent of deficits in vision or cognition [16] and the need for alternate transport was reinforced in our community consultation. In the Hills district, in addition to limited bus routes, two types of community transportation services are available for seniors, firstly social outings in community buses and secondly door-to-door services. The latter run at subsidised cost (i.e., a volunteer gets paid per km to transport the user). The Australian Bureau of Statistics estimates that during a fortnight, those aged 75 and over primarily take trips for shopping (>80%), visiting relatives and friends (58%) but also medical appointments, church or other outings [30]. As part of the intervention delivered in this study, using an occupational therapy problem solving process [59], participants are encouraged to consider life without a licence and devise a plan for staying active, connected and independent within their community when they retire from driving. To formulate this plan, an inventory of desired trips will be taken and the participant counselled about alternatives to driving, utilising public transport, door to door community transport, and council community buses.

### Outcome assessment

#### Safety

A primary aim of this research will be to determine if driving safety is promoted through reduced driving exposure and fewer rapid deceleration events in the group participating in the program. An in-vehicle monitoring device or ‘black box’ will be installed into participants’ own vehicles by a mobile technician. The device consists of an in-vehicle data logger, three-axis accelerometer and GPS receiver, hard-wired to the vehicle which transmits time-stamped data on changes in speed and location every 20 s during vehicle operation. Data will be transferred weekly to a secure server and the devices will remain in the participants’ vehicle for 12 months.

The in-vehicle monitoring system has been validated in pilot study and laboratory experiments [60]. The device also has recently been field tested in 30 vehicles in metropolitan Sydney. Data quality was accurate and validation studies found 97% of trips were captured [61]. Importantly the device requires no involvement from the participant and in previous studies was well received by those involved. Rapid deceleration events will be defined as 500 m-G or higher deceleration [Meredith 2012]. Deceleration has been proposed as an indicator of a possible near–crash event [62]. and is a useful outcome measure when alternative outcomes such as crashes are infrequent and require large sample sizes. In-vehicle monitoring is an objective measure and all data will be processed by research assistants not involved in delivery of the intervention who will be blinded to treatment allocation. As the program is individualised, we hypothesise that the effect of the program will be greater in those participants with worse levels of function and this is a pre-specified sub-group analysis based on scores on the DriveSafe/DriveAware assessment {Kay, 2009 #2230} [48].

Secondary outcome measures of driving exposure include night driving and radius of travel from home. These variables will also be constructed from data collected by the in-vehicle monitoring system. Self-reported driving habits will be used as secondary outcome measures and survey responses compared at the 12-month time-point, adjusting for baseline. We will use an adapted version of the Driving Habits Questionnaire (DHQ) [63] and instruments developed for Australian populations by Baldock et al. [9] and Sullivan et al. [64] to assess self-reported driving habits, confidence in driving and self-reported avoidance of particular driving conditions. Other secondary outcomes include presence of depressive symptoms [65] and mood [34].

#### Mobility

The Keele Assessment of Participation [66] will be administered at baseline and 12 months to assess any change in mobility and community participation in intervention and control groups. This is the primary outcome measure for community participation. Secondary measures of mobility include frequency of socialization, frequency of trips out of the home, both driven and others reported at the 12-month visit. Uptake of alternative forms of transport will also be compared.

### Sample size and statistical analysis

Previous research has found that approximately 12% of older drivers report crash involvement during a 12-month period [67]. However, near misses are approximately 18 times higher than crash rates [68]. We may therefore expect to document approximately 370 incidents (crashes and near misses) during our period of observation (rate 0.73 per person/year)[68]. With a 5% level of significance and 80% power, we require 178 in each arm to detect a 20% reduction in combined crash and near miss rates (i.e., IRR=0.80). Allowing for drop-outs rate of 5%, we will enroll 380 into the program (190 in each arm). We will be able to measure a difference of 6 km in daily distance travelled (~30% reduction), using daily mileage in a population of older drivers [28]. Differences in the Keele Assessment of Participation will also be well served by this sample size.

An intention-to-treat approach will be used for all analyses. Driving exposure (kilometres travelled) and the number of rapid deceleration events (RDE) during 12 months will be compared between the intervention and control groups. A linear regression model will be used to compare the groups with weeks of driving as a covariate. Mobility and community participation will also be compared between the two groups using a similar analytic approach. A subgroup analysis will be performed on each primary outcome defined by the cut-off on DriveSafe/DriveAware [1], for needing further assessment (95 and below). As the primary analysis is based on multiple endpoints (3), the results will be interpreted with caution if the statistical significance is borderline (i.e., a *p*-value between 1.66% and 5%) due to multiplicity issues. Continuous secondary outcomes will be analysed using linear regression models with generalized estimating equations for repeated measures (e.g., weekly radius from home) and count data analysed using Poisson or negative binomial regression models as appropriate. Analyses will be conducted using the SAS and STATA software packages.

### Process evaluation

Measuring intervention implementation in trials has gained momentum in recent decades [69-71] to safeguard against discarding ineffective interventions that were poorly executed or adopting effective interventions that are impractical to implement in the real world [72]. Our intervention, like many complex health interventions, may have several potential ‘active ingredients’ for intervention success or failure [70,73]. By exploring the relationship between intervention outcomes and the quality of intervention implementation, we propose to pinpoint the ‘active ingredients’ responsible for intervention outcomes [70,71,73]. Our process evaluation has three main aims: to measure what is taught (treatment fidelity and dose delivered), what is learnt (dose received), and what is actually used (treatment enactment) [69].

When measuring program fidelity, caution needs to be exercised to avoid overly standardising complex interventions [73,74]. In our intervention, two individualised, one-to-one sessions will address driving self-regulation and planning for retirement from driving. The underlying behavourial theory is standardised, allowing the form of the intervention to be tailored to meet the specific behaviour change needs of participants. There is growing consensus that complex interventions have greater potential for success if they are tailored to the local context [70,73,74].

Using the framework set out by Linnan and Steckler [75] and later extended by Saunders et al. [71], as a guide, the process evaluation will evaluate fidelity, dose (delivered and received), reach, recruitment and context using a combination of qualitative and quantitative data collection methods. Instructor case notes, record of deviations from protocol and intervention checklists will be used to evaluate treatment fidelity and dose delivered and will be processed by a research assistant not involved in delivery of the intervention. Post intervention participant questionnaires administered by a research assistant, not involved in the delivery of the intervention, will be used to measure dose received. Changes to driving exposure and uptake of alternative forms of transport will measure treatment enactment. To evaluate reach and recruitment, all recruitment strategies will be documented and an intervention log recorded for each participant. Data collection for the process evaluation will be embedded within the trial. To prevent interpretation bias, analysis for the process evaluation will be conducted blinded to the intervention group allocation.

## Discussion

Older drivers have high crash involvement and increased vulnerability to injury on the road, however driving is vital to independence for many older Australians. While programs to promote safe mobility are in place, there are few that have been evaluated for their effectiveness. Results from the small number of randomised trials evaluating interventions to promote safety but maintain mobility are mixed [76] and there are no proven strategies to protect older drivers but maintain their mobility [6]. Here we evaluate a safe mobility program which was developed in the United States but adapted for the Australian setting. The program has been adapted in consultation with the community, draws on existing seniors’ mobility services in the local area and employs a validated educational programs for older, at-risk drivers. Therefore the program has the potential to be sustainable. Furthermore the program is based on robust behaviour change theory and delivery of the program will be individualised according to the stages of the precaution adoption model. While there are a number of initiatives from bodies such as motoring associations, local councils and seniors groups, many are not sustained, in part due to the lack of proof of their effectiveness. This randomised trial will provide credible evidence about the efficacy of a safe transport program, using objective measures of driving ability.

The results will have relevance to policy makers and advocates for older members of the community in high income countries internationally, where there is high reliance on private motor vehicles and an ageing population. If proven to be effective, this program could be made available to older members of the community to assist in the timely retirement from driving and continued independent mobility and community engagement. Findings from this research will provide high quality, policy relevant evidence regarding the potential to prevent crash injuries and maintain mobility during the transition to not driving. If proven, this integrated program will help older Australian live productively, independently and safely in the community.

## Competing interests

AB is an author of the Drivesafe Driveaware assessment tool and receives a royalty on sales. There are no other competing interests.

## Authors’ contributions

LK and KC led the methodological design of the study, supported by RI, SB, JB, EC, SL and AB. LK and KC drafted the paper and all authors contributed to revisions. All authors read and approved the final manuscript.

## Pre-publication history

The pre-publication history for this paper can be accessed here:

http://www.biomedcentral.com/1471-2458/13/106/prepub
